# Comprehensive Investigation of Iron Salt Effects on Membrane Bioreactor from Perspective of Controlling Iron Leakage

**DOI:** 10.3390/membranes15100297

**Published:** 2025-09-30

**Authors:** Qiaoying Wang, Bingbing Zhang, Jicheng Sun, Wenjia Zheng, Jie Zhang, Zhichao Wu

**Affiliations:** State Key Laboratory of Water Pollution Control and Green Resource Recycling, Shanghai Institute of Pollution Control and Ecological Security, College of Environmental Science and Engineering, Tongji University, Shanghai 200092, China; qywang@tongji.edu.cn (Q.W.); 2331416@tongji.edu.cn (B.Z.); 1631342@tongji.edu.cn (J.S.); 2130558@tongji.edu.cn (W.Z.); wuzhichao@tongji.edu.cn (Z.W.)

**Keywords:** membrane bioreactor, iron salts, phosphorus removal, leakage

## Abstract

Although adding iron salts can improve phosphorus removal in membrane bioreactor (MBR) processes, overdosing iron salts may result in excessive iron concentrations in the effluent and pose risks of surface water contamination. In this study, an optimized iron salt dosing method was proposed to comprehensively investigate its effects on the performance of MBRs and the control of iron leakage. The results showed that batch dosing of solid iron salts (Fe_2_(SO_4_)_3_) into the influent or activated sludge maintained an effluent Fe^3+^ concentration below 1.0 mg/L and a total phosphorus (TP) concentration below 0.30 mg/L. Long-term operation of the MBR (under conditions of HRT = 4.3 h, SRT = 20 d, and MLSS = 12 g/L) showed that batch dosing of solid iron salts led to an increase in the effluent ammonia–nitrogen (NH_3_-N) concentration, and the nitrification effect was restored after supplementing the alkalinity. Iron salts increased the TP removal rate by approximately 40% while inhibiting the biological phosphorus removal capacity. The average Fe^3+^ concentration in the membrane effluent (0.23 ± 0.11 mg/L) met China’s Environmental Quality Standard for Surface Water (GB3838-2002). This study demonstrates that batch dosing of solid iron salts effectively controls iron concentration in the MBR effluent while preventing secondary pollution. The mechanisms of the impact of iron salts on MBR performance provide crucial theoretical and technical support for MBR process optimization.

## 1. Introduction

With increasingly stringent water quality standards, the membrane bioreactor (MBR) technology has become a research hotspot in the field of water treatment due to its high effluent quality and small footprint [1]. Currently, MBR technology is widely used in municipal and industrial wastewater treatment, as well as in water resource recycling [2,3,4]. However, the MBR process still faces several technical challenges in practical operation. Chief among these is the need for extensive aeration to control membrane fouling, which not only leads to high energy consumption [5] but also inhibits biological phosphorus removal. Thus, chemical phosphorus removal (CPR) using iron/aluminum salts is usually adopted as a supplementary method in MBR processes [6,7].

In the MBR process, coagulants are added to wastewater to form phosphorus-containing solids, and iron salts are among the most commonly used additives in practical projects [8]. Many researchers have also investigated the effect of iron salts on phosphorus removal in MBRs. Li et al. [9] found that when 10, 15, and 20 mg/L ferric chloride (FeCl_3_) were added, the TP removal rates of the MBR were 70.6%, 87.7%, and 96.4%, respectively. Iron salt addition can also effectively mitigate membrane fouling. Yang et al. [10] reported that with a dosage of polymerized ferric chloride (PFC) between 10 and 15 mg/L, the average effluent TP concentration of the MBR reduced to 0.26 mg/L. Previous reports normally focused on the effects of iron salts on TP removal and membrane fouling control, while the leakage of iron salts into the effluent was ignored.

Iron pollution has emerged as a global water contamination issue posing significant potential risks [11]. To meet stricter discharge standards, excessive iron salt dosing for phosphorus removal is widely practiced. However, this approach causes economic losses and poses secondary pollution risks due to residual iron salts in the effluent, which is largely ignored in previous studies. Severe iron pollution in surface and groundwater has been documented, with concentrations reaching 6 mg/L in the Ganges River [12] and widespread exceedances reported elsewhere [13,14]. The influx of Fe^2+^ into aquatic systems facilitates both ferrous sulfide (FeS) formation [15,16] and P release from sediments [17], which intensifies hypoxia and eventually leads to black odor [18,19]. Conventional drinking water treatment processes exhibit limited efficacy in dissolved iron removal. Chronic exposure to iron-contaminated water may induce severe health consequences, including hepatotoxicity, neurodegenerative disorders, and carcinogenic effects [20,21]. Zhuang et al. [22] demonstrated that iron salts readily combine with perfluorinated compounds, subsequently enhancing disinfection by-product formation and compromising water quality safety. Therefore, optimizing iron salt dosing to maintain effluent iron below the 0.3 mg/L threshold specified in China’s Environmental Quality Standard for Surface Water (GB 3838-2002) and elucidating the iron retention mechanisms of microfiltration membranes represent research priorities for MBR enhancement.

In a MBR system, iron salt dosing alters mixture characteristics and increases phosphorus removal complexity [23]. Optimizing iron salt dosing requires balancing effluent iron levels, membrane fouling mitigation, and microbial activity maintenance by optimizing key parameters, including dosing method, concentration, and reaction conditions. Gkotsis et al. [24] reported that continuous coagulant dosing achieved a 40% greater TMP reduction compared to intermittent dosing. Li et al. [25] determined that 1.8 mmol/L FeCl_3_ provided optimal performance with 96.7% TP removal, 21.9% lower TMP, and stable TN removal. However, higher doses impaired PAOs and AOB activity, reducing AOB activity by 60.7%. Existing studies have focused more on the impact of dosing methods on membrane fouling and phosphorus/nitrogen removal, while neglecting the critical indicator of effluent iron concentration.

In contrast to previous studies, this research considers effluent iron concentration as a core evaluation indicator. A systematic comparison of three dosing strategies was conducted: one-time dosing of solid iron salts, continuous dosing of iron salt solution, and batch dosing of solid iron salts. The aim was to identify an optimal approach that ensures MBR performance while minimizing secondary pollution risk. The long-term impacts of the optimal dosing method on effluent quality, membrane fouling, and microbial communities were further evaluated, with emphasis on monitoring effluent iron concentration to assess environmental risks. The results demonstrated that iron salt dosing enhanced TP removal while inhibiting the nitrification process (reversible with alkalinity addition), and appropriate dosing strategies effectively controlled residual iron levels. This study provides a new perspective for the green and sustainable development of MBR technology by achieving synergistic optimization of effluent iron control and water quality improvement. It also offers important insights for addressing iron pollution as a global water environmental issue.

## 2. Materials and Methods

### 2.1. Materials

Ferric sulfate (Fe_2_(SO_4_)_3_, 21–23% Fe content) and 1,10–phenanthroline were analytical reagent grade and purchased from Aladdin Biotechnology (Shanghai, China). The polyvinylidene fluoride (PVDF) microfiltration membrane used in this study, with an average pore size of 0.2 µm, was prepared in the laboratory. The properties of the PVDF membrane are provided in our previous report [26,27,28]. The domestic wastewater used in the continuous flow experiment was collected from the effluent of the sand sedimentation tank of the Quyang Wastewater Treatment Plant (WWTP) in Shanghai, China. The key characteristics of the domestic wastewater were as follows: COD: 350 ± 94 mg/L, NH_3_-N: 32.8 ± 4.6 mg/L, TN: 41.6 ± 3.7 mg/L, TP: 4.4 ± 0.6 mg/L. The detailed water quality of the influent during the test period is shown in Table S1. The activated sludge was collected from a laboratory MBR unit that had not been exposed to iron salts. For the long-term operation experiment, the MBR was inoculated with activated sludge from the aerobic tank of the WWTP. The influent quality was identical to the domestic wastewater described above.

### 2.2. Experimental Methods

#### 2.2.1. Continuous Flow Experiments

A schematic diagram of the continuous flow experimental setup is shown in Figure S1. The size of the reactor was L × B × H = 6 × 6 × 15 cm with a volume of 0.5 L. A flat-sheet microfiltration membrane module with an effective filtration area of 48 cm^2^ was installed in the reactor. Baffles were installed on both sides of the membrane module to form the up-flow zone, with a spacing of 1.0 cm between the baffles and the module. The membrane flux was set to 20 L/m^2^·h (LMH), and the hydraulic retention time (HRT) was 4.0 h.

Three Fe_2_(SO_4_)_3_ dosing methods were set up to investigate their effects on iron leakage and TP removal, and the running time varied according to different test conditions (mainly 4 h and 24 h). The three dosing methods are shown below: (1) One-time dosing of solid iron salts: The total amount of solid iron salts required for chemical phosphorus removal over the entire test duration was added at once. (2) Continuous dosing of iron salt solution: First, a certain concentration of iron salt solution was prepared. To prevent hydrolysis and ensure stability [29,30], H_2_SO_4_ was added to maintain the pH below 2.0, a standard laboratory procedure that would cause a transient decrease in the pH within the reactor. Finally, the solution was dosed at a controlled rate using peristaltic pumps. (3) Batch dosing solid iron salts: A certain time interval (4 h in the test, equal to 1 HRT) dosing iron salt solids required for chemical phosphorus removal in the corresponding time.

#### 2.2.2. MBR Long-Term Operation Test

The associated designs of two sets of MBR set-ups used in this study are shown in Figure S2, and the design parameters are shown in Table S2. The effective volume was 10.5 L, and the aeration intensity was 1.0 m^3^/m^2^·min. To ensure effective aeration, the bottom aeration pipe was positioned 500 mm from the bottom of the membrane modules. The designed operational parameters were as follows: sludge retention time (SRT), 20 d; mixed liquor suspended solids (MLSS) concentration, 12 g/L; membrane flux, 20 LMH; hydraulic retention time (HRT), 4.3 h. The reactor operation mode was “pump 10 stop 2”.

The reactor with iron salt dosing was numbered as reactor #1, while the undosed reactor served as the control (reactor #2). Iron salts were dosed daily at approximately 9:00 at a molar Fe/P ratio of 1.5, corresponding to approximately 64 mg/L of Fe_2_(SO_4_)_3_. The calculation procedure is provided in the Supporting Information (Text S1). The experiment comprised three stages: Stage 1: Both MBR systems were operated to reach a stable state. Stage 2: Iron salts were added to reactor #1, while reactor #2 still maintained original operating parameters as the control. Stage 3: Dosing was discontinued in reactor #1, while #2 remained unchanged.

### 2.3. Analytical Methods

#### 2.3.1. Routine Chemical Test Items and Methods

The details of routine chemical test items and methods are provided in the Supporting Information (Table S3). Effluent quality was assessed according to the Grade A criteria specified in the Chinese Discharge Standard of Pollutants for Municipal Wastewater Treatment Plant (GB 18918-2002). The specific limit values are listed in Table S4. Rigorous quality control was implemented throughout the study, including the use of standard calibration curves (R^2^ > 0.999), analysis of parallel samples, and routine blank tests.

#### 2.3.2. Method for the Determination of Fe^3+^

Fe^3+^ concentrations were determined using the o-phenanthroline colorimetric method. The detection limit of this method was 0.005 mg/L. The procedure was as follows: (1) Total iron, 1 mL of the solution was pipetted into a 25 mL colorimetric tube. Then, 1 mL of reducing agent (10% hydroxylamine hydrochloride), 1 mL of pH buffer (NH_4_Ac-HAc), and 1 mL of color reagent (0.1% o-phenanthroline) were added sequentially. The volume was adjusted to 25 mL, shaken, and absorbance was measured at 510 nm after 5 min. (2) Fe^2+^: The procedure was the same as (1) except that the hydroxylamine hydrochloride was omitted. (3) Samples with flocculation or precipitation: 50 mL of the water sample was added to a 250 mL conical flask. A few drops of HCl (1:1) were added to adjust the pH to <2, followed by heating and boiling for 10 min. After cooling, it was transferred to a 50 mL volumetric flask, and deionized water was added to the mark. The solution was shaken well, and 1 mL was pipetted into a 25 mL colorimetric tube following the procedure outlined in step (1). (4) Fe^3+^: The Fe^3+^ concentration was calculated by subtracting the Fe^2+^ concentration from the total iron concentration.

#### 2.3.3. Classification of Different Forms of Fe^3+^

Following the method of Wang et al. [31], iron ions in the solution were divided into three categories using filters with different pore sizes: (1) Total iron (M_1_): The total iron content was directly determined from the test sample using the method described in 2.3.2. (2) Particulate Fe^3+^ (M_3_): The test sample was passed through a 0.80 μm filter, and the Fe^3+^ content of the filtrate, M_2_, was measured. The difference between M_1_ and M_2_ was the particulate Fe^3+^ content M_3_. (3) Dissolved Fe^3+^ (M_4_): The sample was passed through a 0.22 μm filter, and the Fe^3+^ content of the filtrate, M_4_, was measured. (4) Colloidal Fe^3+^ (M_5_): The difference between M_2_ and M_4_ was the content of colloidal Fe^3+^ M_5_.

#### 2.3.4. Determination of Phosphorus Release and Phosphorus Absorption Capacity

The phosphorus release rate from activated sludge was tested in a sealed 1 L reactor (Figure S3) under anaerobic conditions, with MLSS controlled to 3–4 g/L. During the test, sludge mixture samples were taken every 10 min and immediately filtered with a 0.45 μm filter. After 1 h, the sampling interval was increased to 20 min, and the total sampling time was 3 h. The soluble orthophosphate concentration in the filtrate was measured immediately, and the phosphorus release rate (PRR) was determined from the slope of its linear time-dependent plot. The specific phosphorus release rate (SPRR) was calculated as the ratio of PRR to MLVSS.

The activated sludge phosphorus absorption rate was measured immediately after the anaerobic phosphorus release process using the device shown in Figure S4. The sludge mixture was aerated through the sand core aeration head to maintain a suspended state, with DO controlled at 6–8 mg/L. The sludge mixture was taken every 10 min (adjusted to 20 min after 1 h), and the sampling time was 3 h in total, with immediate filtration through 0.45 μm membranes. The phosphorus uptake rate (PUR) was determined from the slope of the straight line between filtrate orthophosphate concentration and time. The specific phosphorus uptake rate (SPUR) was calculated as the ratio of PUR to MLVSS.

#### 2.3.5. Determination of Sludge-Specific Nitrification Rate

The specific nitrification rate was measured using the same setup as for the SPUR. The specific test method was as follows: A volume of activated sludge was centrifuged (3000 r/min, 5 min). After discarding the supernatant, the remaining sludge solids were resuspended in PBS (pH 7.0). The sludge concentration was adjusted to 2–3 g/L, supplemented with 100 mg/L NaHCO_3_ and 76 mg/L NH_4_Cl to control the initial NH_3_-N concentration at about 20 mg/L, and aerated to maintain 6–8 mg/L DO. Samples of the sludge mixture were collected every 20 min over a 3 h period. The sludge mixture was filtered, and the NH_3_-N concentration of the filtrate was measured. The NH_3_-N values and time were plotted to generate a straight line. The absolute value of the slope was determined as the nitrification rate (AUR) of the sludge, and the ratio of AUR to MLVSS was calculated as the specific nitrification rate (SAUR).

#### 2.3.6. Determination of Sludge-Specific Oxygen Uptake Rate

The method for determining the specific oxygen uptake rate (SOUR) and the experimental setup are detailed in the Supplementary Information (Text S2, Figure S5).

#### 2.3.7. Testing of Molecular Weight Distribution of Organic Matter

Gel filtration chromatography (Le-10ADVP, Shimadzu) was used to determine the molecular weight distribution of organics in water. Prior to analysis, samples were filtered through a 0.45 μm syringe filter and sonicated to remove air bubbles.

#### 2.3.8. Statistical Analysis

Statistical analyses were conducted using IBM SPSS Statistics 27. Data are expressed as mean ± standard deviation (mean ± s.d.). Differences between the two groups were assessed using independent samples t-tests, paired sample t-tests, or Mann–Whitney U tests. Correlation analysis was used to evaluate associations between variables. *p* < 0.05 was considered significant.

## 3. Results and Discussions

### 3.1. Optimization of Iron Salt Dosing in MBR

#### 3.1.1. Optimization of Iron Salt Addition in Influent

**Figure 1:**
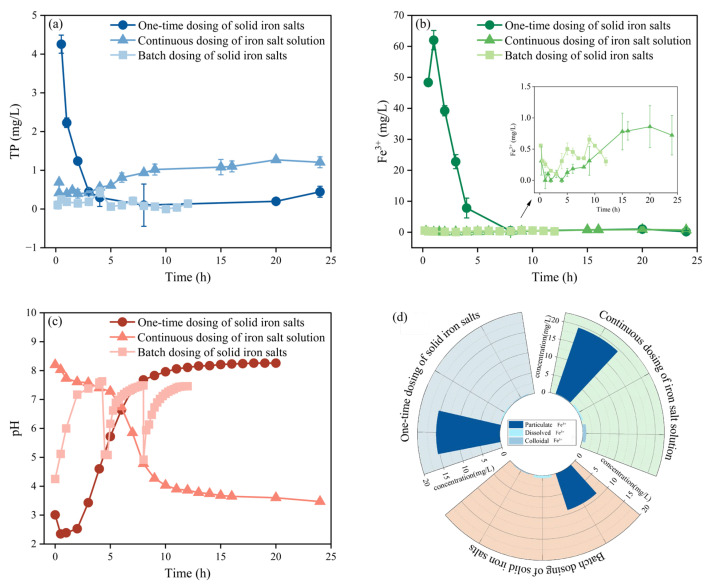
Effects of iron salt dosing in influent on (**a**) TP, (**b**) Fe^3+^, and (**c**) pH; (**d**) concentrations of particulate Fe^3+^ (>0.8 μm), dissolved Fe^3+^ (<0.22 μm), and colloidal Fe^3+^ (0.22–0.80 μm) in wastewater under three dosing methods; data in (**a**,**b**) are presented as mean ± standard deviation (*n* = 3).

As shown in Figure 1a, after one-time dosing of solid iron salts to the influent, the TP concentration in the effluent decreased to 0.50 mg/L after 3 h and reached 0.45 mg/L (with a removal rate of 91.3%) after 24 h. Continuous dosing of iron salt solution resulted in an effluent TP concentration of 0.42 mg/L at 0.3 h, which increased to 1.21 mg/L after 24 h, showing a removal rate of only 72.9%. Batch dosing of solid iron salts achieved a 97.48% TP removal rate within 10 min (0.10 mg/L effluent) after the first dosing. Although TP concentration slightly increased to 0.44 mg/L after 4 h, the average TP concentration was 0.09 ± 0.06 mg/L during 5–12 h, with an average removal rate of 97.8%.

As shown in Figure 1b, one-time dosing resulted in effluent Fe^3+^ concentration peaking at 62.01 mg/L after 1 h, decreasing to 7.82 mg/L after 4 h, and stabilizing at approximately 0.50 mg/L after 8 h. As shown in Figure 1d, Fe^3+^ in the reactor primarily existed in particulate form (15.45 mg/L, 96.6%) after 24 h, while colloidal and dissolved states had lower concentrations. The flat-sheet microfiltration membrane effectively retained the particulate Fe^3+^, reducing effluent concentration. The continuous dosing initially yielded low effluent Fe^3+^ (0.78 mg/L at 15 h), then stabilized around 0.90 mg/L. Although maintaining Fe/P = 1.5, Fe^3+^ gradually accumulated and was discharged due to slightly excessive dosage. Fe^3+^ in the reactor was still primarily in particulate form (19.45 mg/L, 93.1%) after 12 h (Figure 1d). When solid iron salts were added in batches, the Fe^3+^ concentration exhibited periodic fluctuations, with a rapid increase at the beginning of each addition, but overall remaining below 1.00 mg/L, followed by a decrease. Fe^3+^ in the reactor primarily existed in particulate form (9.04 mg/L, 92.1%) after 12 h.

As shown in Figure 1c, after one-time dosing, the system pH first decreased to 2.3 (0.5 h) and then gradually increased to 8.1 (11 h). The hydrolysis of the large quantity of added solid iron salts acidified the solution. The low pH inhibited the hydrolysis of iron salts into flocs or precipitates, reducing P removal and the loss of dissolved iron salts. A subsequent increase in pH improved P removal, but as iron salts were consumed, TP concentration rose again, requiring timely replenishment of iron salts. The continuous dosing induced a strongly acidic environment (pH 3.5). This was attributed to both the accumulation and hydrolysis of iron salts, as well as the inherent acidity of the iron salt solution being dosed. Consequently, the effects observed under this dosing mode resulted from the combination of these factors. The batch dosing induced minor pH fluctuations, remaining above 4.0 initially and slowly rising to approximately 8.2 after 4 h. Following each addition, the pH exhibited periodic fluctuations and rapidly recovered to above 8.0, a condition favorable for P removal by iron salts.

#### 3.1.2. Optimization of Iron Salt Dosing in Activated Sludge

**Figure 2:**
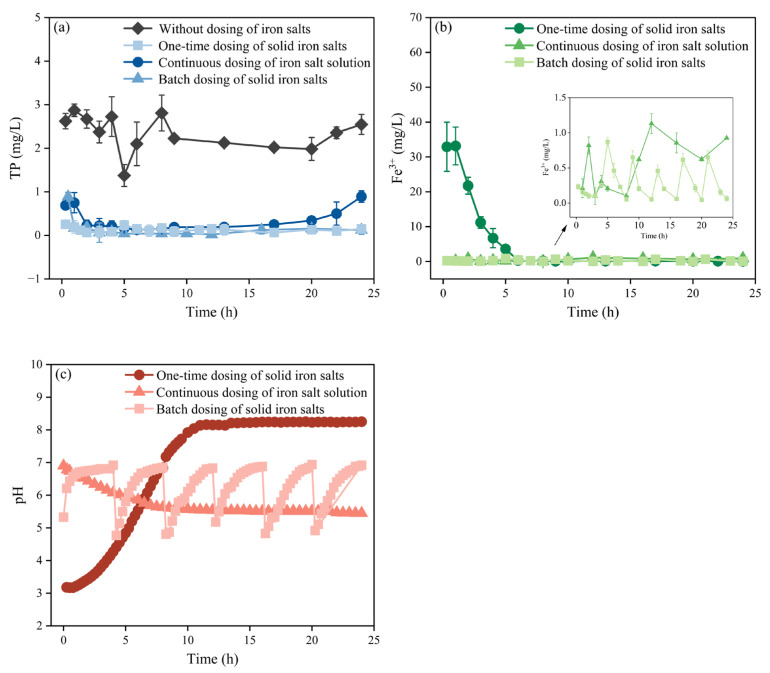
Effects of iron salt dosing in activated sludge on (**a**) TP, (**b**) Fe^3+^, and (**c**) pH; data in (**a**,**b**) are presented as mean ± standard deviation (*n* = 3).

In this experiment, the control MBR system without dosing of iron salts was set. The MBR system achieved TP removal, which was inefficient due to the lack of an anaerobic section. As shown in Figure 2a, the control MBR system exhibited poor TP removal performance, with an average effluent TP concentration of 2.34 ± 0.23 mg/L (45.2% removal). The one-time dosing rapidly reduced the effluent TP to below 0.30 mg/L within 2 h but gradually rebounded to 0.89 mg/L after 24 h. In contrast, the continuous dosing resulted in superior P removal. The effluent TP decreased to 0.88 mg/L within 0.5 h, stabilized below 0.30 mg/L, and reached 0.12 mg/L after 24 h, achieving a removal rate of 97.1%. The batch dosing maintained the effluent TP concentration below 0.30 mg/L. Although slight periodic fluctuations were observed, the levels remained stable overall, with an average concentration of 0.14 ± 0.06 mg/L and a removal rate of 97.7%.

As shown in Figure 2b, effluent Fe^3+^ concentration exhibited distinct patterns under different dosing strategies. The one-time dosing caused an initial Fe^3+^ concentration peak (32.91 mg/L) after 1 h, then gradually decreased to 0.31 mg/L at 6 h. The average concentration from 6 to 24 h was 0.14 ± 0.08 mg/L, maintaining a low level. The continuous dosing maintained Fe^3+^ concentration below 1.0 mg/L overall, despite a slight increase in the later stages, with an average concentration of 0.54 ± 0.37 mg/L. Under batch dosing, the Fe^3+^ changed periodically, synchronized with the dosing times. It increased rapidly, immediately after each addition, but remained below 1.0 mg/L overall before declining.

The activated sludge system exhibited greater pH stability than the influent due to abundant microorganisms and buffering substances. As shown in Figure 2c, the one-time dosing caused an initial pH drop to 3.2, then gradually rose to approximately 8.2 due to iron salt efflux and wastewater inflow, and remained stable. Continuous dosing slowly acidified the system to a pH of 5.4, above the normal microbial activity threshold (pH > 5.0) [32], resulting in a minimal impact. In activated sludge systems with sufficient buffering capacity, it is more applicable than dosing directly into the influent. However, the acidic environment it creates may still pose risks to long-term system stability. Therefore, implementing accompanying pH adjustment measures may be necessary in practical applications. The batch dosing showed minimal pH fluctuations, with minimal initial decline and rapid recovery, ultimately stabilizing at 6.8–6.9. This is beneficial for P removal and reduces iron salt loss.

Batch dosing of solid iron salts avoids the high initial Fe^3+^ concentration associated with one-time dosing and mitigates Fe^3+^ accumulation from continuous dosing. Consequently, it achieves improved P removal alongside stable and low effluent Fe^3+^ levels. This mechanism is applicable to various wastewater sources. However, the efficiency of P removal, microbial activity, and iron concentration in the effluent are influenced by wastewater composition and operational conditions. For industrial wastewater with extremely low pH [33], the hydrolysis and precipitation behavior of iron may be altered, necessitating adjustments to the dosing frequency or supplemental pH control. High concentrations of complexing [34] or chelating agents (e.g., EDTA, citrate) may form stable soluble iron complexes, which reduce P removal and increase iron leakage risk. Nevertheless, the batch dosing strategy plays a critical role in controlling iron leakage and provides a reference for a wide range of applications.

### 3.2. Effect of Iron Salts on Pollutant Removal in MBR Systems

#### 3.2.1. Water Quality Analysis

To enable a parallel comparison between the two devices, the effluent quality and operating conditions were kept consistent during the 75 d stabilization period. As shown in Table 1, after an initial 25 d acclimation period, both MBR systems achieved consistent effluent quality (25–75 d), enabling further comparative analysis. As only the aerobic section was set up in the system, the nitrogen and phosphorus removal effects were general, and the TN in the effluent water mainly existed in the form of NO_3_-N.

**Figure 3:**
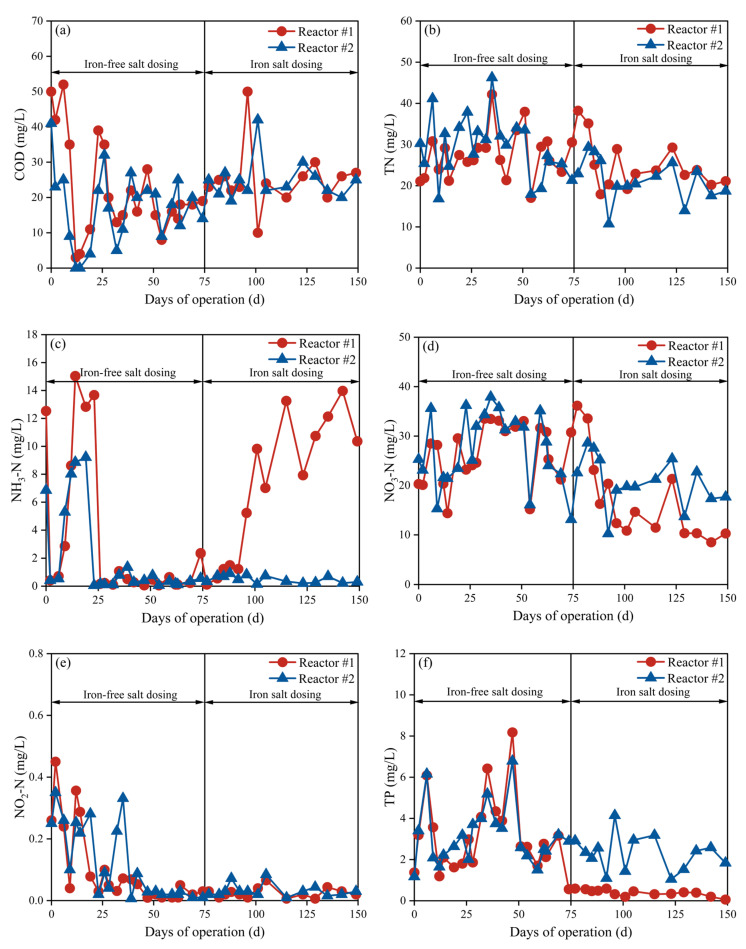
Variations in water quality during operation of reactors #1 and #2: (**a**) COD; (**b**) NH_3_-N; (**c**) TN; (**d**) NO_3_-N; (**e**) NO_2_-N; (**f**) TP.

At approximately 75 days, Fe_2_(SO_4_)_3_ was added to reactor #1, while reactor #2 maintained its original operating conditions as the control. The effluent quality from both systems is shown in Figure 3. As shown in Figure 3a, effluent COD levels in both reactors remained low and showed no significant difference (p = 0.474). This result contrasts sharply with literature reports of an iron salt-enhanced coagulation effect in conventional activated sludge systems. This discrepancy originates from the MBR’s inherent microfiltration-level retention effect, which achieves good solid–liquid separation performance without relying on chemical coagulation.

#### 3.2.2. Nitrogen Removal

Both systems showed limited denitrification capacity, owing to the absence of an anoxic functional zone. As shown in Figure 3b, the effluent TN concentrations showed no significant difference (*p* = 0.919) between the two reactors (#1: 25.27 ± 5.96 mg/L; #2: 21.36 ± 5.03 mg/L). However, as shown in Figure 3c, reaction #1 exhibited a slowly increasing trend in effluent NH_3_-N after iron salt dosing, ultimately exceeding the 10 mg/L threshold during later operational stages, while the control group maintained at 0.50 ± 0.26 mg/L. Figure 3d reveals an inverse trend for NO_3_-N, with reaction #1 exhibiting 11% lower concentration (18.02 ± 9.20 mg/L) compared to the control. This indicates that iron salts might inhibit the microbial nitrification process.

Previous studies have indicated that iron salts impact nitrification through multiple mechanisms. The structure formed by iron and phosphates enhances the compactness of sludge flocs, hindering the absorption and transfer of nutrients like NH_4_^+^-N, and limiting oxygen contact with nitrifying bacteria, thereby reducing nitrification efficiency [35,36]. Additionally, when the pH is < 6.0, the NH_3_-N concentration in MBR effluent is difficult to stabilize below 1.0 mg/L [23]. Excessive iron salt introduction can lower system pH, inhibiting nitrifying bacteria activity [37]. This further confirms the inhibitory effect of iron salts on nitrification.

**Figure 4:**
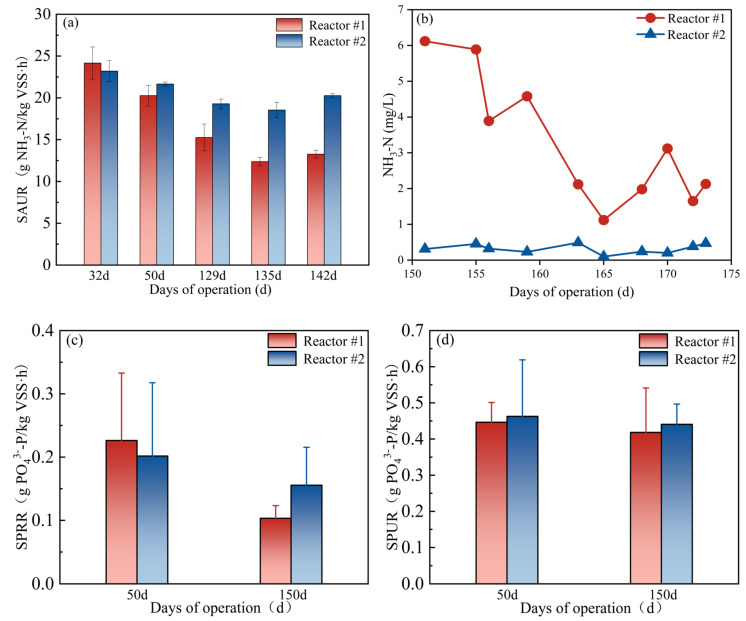
Comparison between reactors #1 and #2: (**a**) SAUR; (**b**) changes in NH_3_-N concentration in the effluent after adjusting alkalinity; (**c**) SPRR; (**d**) SPUR. Data in (**a**,**c**,**d**) are presented as mean ± standard deviation (*n* = 3).

This study further revealed the influence of iron salts on nitrification performance by comparing the SAUR of two devices. Figure 4a demonstrated that while the initial SAUR values were comparable between the two reactors, continuous iron salt dosing in reaction #1 caused significant SAUR reduction (*p* = 0.002), declining to 13.26 g NH_3_-N/(kg VSS h) by day 142. This value was considerably lower than the 20.27 g NH_3_-N/(kg VSS h) of reaction #2. The iron salts dosage showed a significant inverse correlation with SAUR (*p* = 0.005). These results indicate that iron salts directly reduce the nitrification rate, resulting in NH_3_-N accumulation, while NO_3_-N decreases in reactor #1 effluent. These findings are consistent with previous literature that iron salts inhibit nitrification.

According to the stoichiometry of nitrification, oxidizing one unit of NH_3_-N to NO_3_^-^ consumes 4.57 units of oxygen and 7.14 units of alkalinity (calculated as CaCO_3_). The denitrification theoretically can compensate for approximately 50% of nitrification’s alkalinity demand. However, the absence of an anoxic zone in this system precluded denitrification, making influent alkalinity the sole source of replenishment. Alkalinity tests revealed lower levels in reaction #1 (92 ± 31 mg/L) than in reaction #2 (178 ± 26 mg/L), indicating that iron salts consumed approximately 48.3% of the system’s alkalinity. Theoretical calculations indicate that complete nitrification of the influent NH_3_-N (~20 mg/L) to an effluent NH_3_-N ≤ 1 mg/L would require 136 mg/L alkalinity. However, the measured alkalinity in reaction #1 only supplies 67.6% of this theoretical demand, directly explaining its low nitrification efficiency. As shown in Figure S6, the SOUR values of reactors #1 and #2 showed little difference and both remained within the normal range for microbial metabolic activity. This indicated that iron salt addition did not inhibit overall microbial activity, thereby ruling out DO limitation as the primary cause of nitrification inhibition.

To verify that alkalinity consumption was the dominant mechanism inhibiting nitrification and to improve performance, CaO (35 mg/L) was added to reaction #1 from day 151 to replenish alkalinity. As shown in Figure 4b, this adjustment significantly reduced effluent NH_3_-N concentration (*p* = 0.001) and improved nitrification performance. This result confirms that alkalinity is a key factor limiting nitrification efficiency and clarifies that the inhibition caused by iron salts is reversible, primarily through the pH decrease induced by iron hydrolysis. This finding provides important practical evidence for the synergistic optimization of iron salt phosphorus removal and biological denitrification in MBR systems.

#### 3.2.3. TP Removal

As shown in Figure 3f, the addition of iron salts reduced the effluent TP concentration in reactor #1 to 0.40 ± 0.16 mg/L, meeting the Grade A discharge standard (GB 18918-2002). The average concentration of TP in the effluent of reactor #2 was 2.33 ± 0.85 mg/L, and the contribution of iron salts to the removal of TP in the system reached about 40%.

To investigate the effect of iron salts on the microbial phosphorus removal capacity, activated sludge was sampled from both reactors at the beginning and end of operation. Their phosphorus release and uptake capacities were expressed as SPRR and SPUR, respectively, with the results shown in Figure 4c,d. At 50 d, both reactors were in the late stage of sludge acclimatization prior to iron salt dosing. The activated sludge in both reactors exhibited similar properties, with SPRR values of 0.23 and 0.20 g PO_4_^3−^-P/(kg VSS·h) for reactors #1 and #2, respectively, and corresponding SPUR values of 0.44 and 0.46 g PO_4_^3−^-P/(kg VSS·h). At 150 d, after 76 days of iron salt addition to reactor #1, its SPRR and SPUR values had decreased to 0.10 and 0.41 g PO_4_^3−^-P/(kg VSS·h), respectively. For a contemporaneous comparison to eliminate temperature effects, reactor #2 maintained SPRR and SPUR values of 0.15 and 0.44 g PO_4_^3−^P/(kg VSS·h), respectively, during the same period. The prolonged iron salt dosing in reactor #1 led to a decline in biological phosphorus removal capacity, attributable to a reduction in the relative abundance of known phosphorus-colonizing bacteria [38].

**Figure 5:**
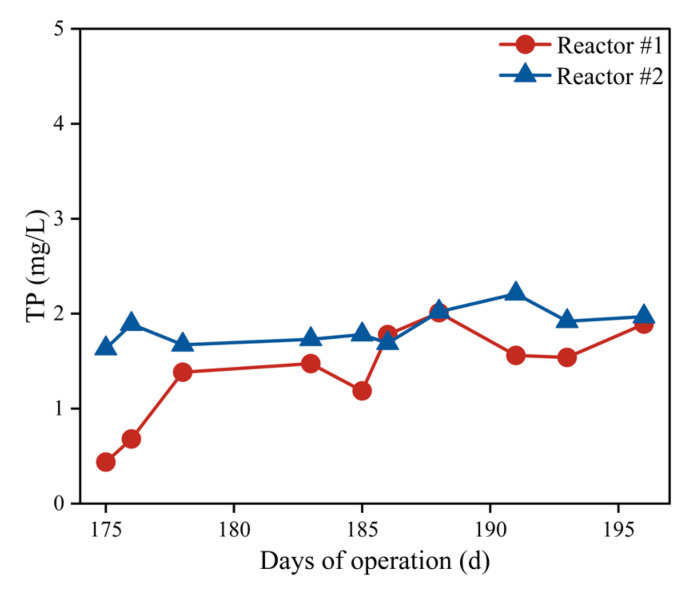
Variations of TP concentration in the effluent from reactors #1 and #2 after ceasing the addition of iron salts.

To investigate iron salts’ impact on microbial phosphorus removal capacity, dosing was ceased in reactor #1 at 175 d, with subsequent effluent TP concentrations in both reactors shown in Figure 5. The effluent TP concentration remained stable in reactor #2, while reactor #1 initially maintained a low TP concentration following dosing cessation. This may be due to residual Fe^3+^ and their complexes adsorbing phosphate. Although phosphorus concentrations later rose to become consistent with reactor #2, no deterioration beyond the control level occurred. This can be attributed to the effective retention of particulate phosphate by the microfiltration membrane.

### 3.3. Contribution of Membrane Retention to Effluent Water Quality

**Figure 6:**
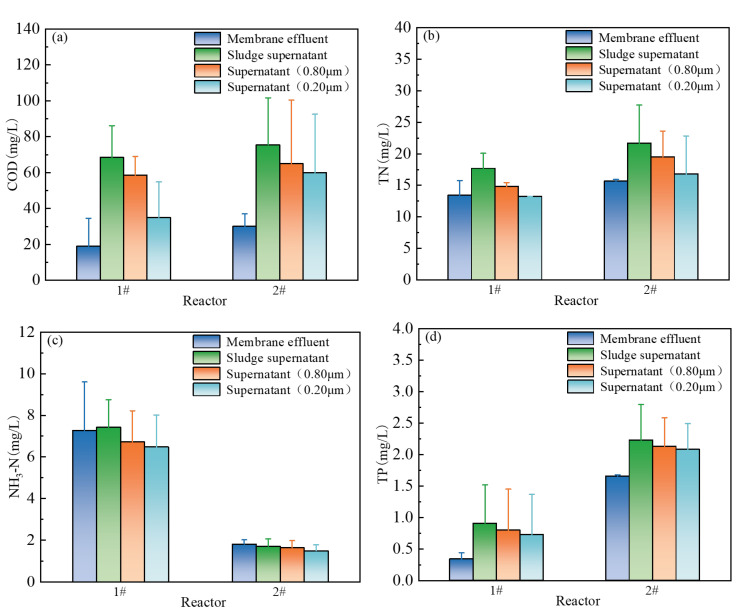
Comparison of membrane effluent, supernatant, and filtrate water quality between reactions #1 and #2: (**a**) COD; (**b**) NH_3_-N; (**c**) TN; (**d**) TP. Data in (**a**,**b**) are presented as mean ± standard deviation (*n* = 3).

This study utilized a multi-stage membrane separation technology system to investigate microfiltration membrane’s retention characteristics in an iron-enhanced MBR process and its regulatory mechanisms on effluent water quality. Water samples were collected from the membrane effluent, the supernatant of the sludge mixture, and the filtrates obtained by passing the supernatant through 0.80 μm and 0.22 μm membranes. Key water quality indicators were analyzed. The results are shown in Figure 6.

A comparative analysis of these water samples (Figure 6a) revealed that the COD concentration in the membrane effluent was lower than that in the supernatant and even the 0.22 μm filtrate. These results demonstrate that the smaller pore size of the membrane effectively retained particulate pollutants, while the sludge layer provided additional organic matter filtration and adsorption, collectively enhancing COD removal. The slightly lower COD values in both the supernatant and effluent of reaction #1 compared to reaction #2 indicate that iron salts reduced the dissolved organic matter content.

Regarding TN, Figure 6b shows a lower concentration in the membrane effluent than in the sludge supernatant. Additionally, the TN concentration in the effluent from different pore sizes also showed a decrease, confirming the microfiltration membrane’s retention of particulate nitrogen as a key contributor to enhanced TN removal. Figure 6c revealed comparable NH_3_-N concentrations in the membrane effluent and the supernatant, indicating that NH_3_-N removal primarily relied on sludge nitrification rather than membrane retention or adsorption.

As shown in Figure 6d, the higher TP concentrations in the filtrates compared to the membrane effluent indicated the microfiltration membrane’s retention and adsorption of colloidal and particulate phosphorus in the mixed liquor. Comparison of the TP concentrations in the effluent and supernatant of reactions #1 and #2 further confirmed that iron salt dosing enhanced P removal. This enhancement occurred through two synergistic mechanisms: (1) physical–chemical precipitation of phosphate by iron salts into larger particles; and (2) effective retention of these particles by the microfiltration membrane. This study reveals that the microfiltration membrane selectively retains colloids (0.22–0.80 μm) and elucidates the synergistic purification mechanism between iron salt-modified sludge flocs and the microfiltration membrane.

### 3.4. The Effect of Iron Salt Addition on Membrane Fouling

**Figure 7:**
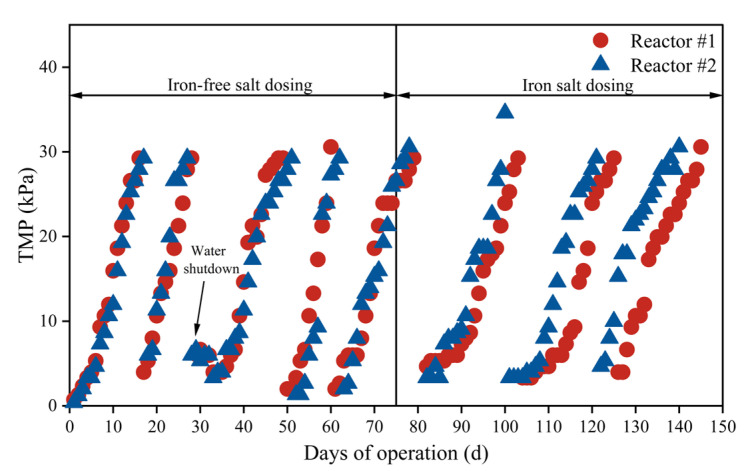
Comparison of TMP between reactors #1 and #2 during operation.

TMP variations between reactors #1 and #2 were analyzed to assess the effect of iron salt addition on membrane fouling. Figure 7 shows that while both reactors initially exhibited similar TMP trends, reactor #1 exhibited a slower TMP increase rate after iron salt addition. At the 30 kPa pressure endpoint, reactor #1 exhibited delayed fouling progression compared to reactor #2, extending its average operating cycle from 19.0 d to 21.6 d. These observations preliminarily suggest that iron salts may contribute to the mitigation of membrane fouling.

The reduced membrane fouling rate may be attributed to the coagulation and removal of macromolecules by iron salts. The supernatants from both reactors, filtered through 0.45 μm membranes, were analyzed by gel filtration chromatography (GFC) to test the molecular weight distribution (Table 2). The GFC analysis revealed three characteristic peaks (P1–P3) in the supernatant of both reactors, with reactor #1 exhibiting lower Mn values (70.1, 336.3, and 2722.9 kDa) compared to reactor #2 (79.2, 361.0, and 4905.8 kDa). The organic compounds in both reactors showed comparable molecular weights between 104 and 105 Da, but reactor #1 exhibited significantly lower molecular weight than reactor #2 when the molecular weight exceeded 106 Da.

The MBR process contains higher levels of high-molecular-weight (>104 Da) microbial metabolites (such as SMP and EPS) compared to traditional wastewater treatment systems [39]. It is speculated that the iron salts added to reactor #1 removed macromolecules through coagulation and adsorption, thereby reducing their concentration in the sludge mixture. The lower supernatant COD concentration in reactor #1 than in reactor #2 (Figure 6a) indicated a reduced macromolecular content. These macromolecular and recalcitrant organic compounds are well-documented as significant membrane foulants [40,41].

### 3.5. Changes in Fe^3+^ Concentration in Membrane Effluent

**Figure 8:**
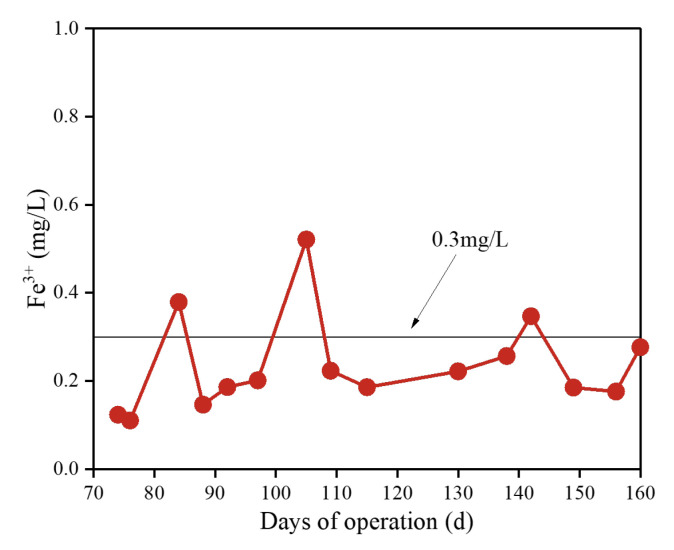
Variations of Fe^3+^ concentration in effluent during iron salt dosing.

After adding iron salts to reactor #1, membrane effluent samples were regularly collected at approximately 1 h post-dosing for Fe^3+^ concentration analysis, with results presented in Figure 8. The figure shows that reactor #1 maintained a relatively low Fe^3+^ concentration (0.23 ± 0.11 mg/L) over approximately 100 d of iron salt addition. The effluent Fe^3+^ concentration complied with China’s Environmental Quality Standard for Surface Water (GB 3838-2002) (≤0.30 mg/L), indicating a low environmental risk. Wang et al. [31] reported that effluent Fe^3+^ concentration accounted for only about 10% of that in the 0.2 μm filtrate supernatant, suggesting that fine-scale Fe^3+^ may be retained in the system. Furthermore, the added Fe^3+^ can form monomeric complexes or amorphous ferric oxide (AFOs) [42] with macromolecules like SMP, which are retained in the sludge mixed liquor or on the membrane surface. Concurrently, larger iron salt particles were effectively filtered by the microfiltration membrane, maintaining a low effluent Fe^3+^ concentration.

Figure 8 also shows periodic fluctuations in effluent Fe^3+^ concentration, with occasional spikes slightly exceeding the 0.30 mg/L limit. Based on cleaning data, this phenomenon primarily occurred immediately after membrane cleaning. Li et al. [43] reported that approximately 98% of system Fe^3+^ was retained in the sludge mixed liquor with minimal distribution in the cake and gel layer. This suggests that the established fouling layer adsorbs iron salts, whereas newly cleaned membranes had not yet formed a significant cake and gel layer, leading to the release of some dissolved Fe^3+^ into the effluent. In Figure 2b, the higher initial effluent Fe^3+^ concentration in the short-term test (24 h) compared to reactor #1 resulted from the membrane surface not having yet formed a fouling layer, further validating the adsorption effect of the fouling layer on Fe^3+^.

## 4. Conclusions

This study proposes an optimized iron dosing strategy for MBR systems that ensures efficient phosphorus removal while minimizing iron consumption. Batch dosing of solid iron salts maintained the Fe^3+^ concentration below 1.0 mg/L while achieving 97% TP removal. Long-term operation revealed that iron salt dosing caused effluent NH_3_-N exceeding 10 mg/L, but this was effectively mitigated by CaO supplementation. While iron salts contributed approximately 40% to TP removal, SPRR and SPUR data indicated partial inhibition of biological phosphorus removal capacity. The results indicated that iron salts preliminarily mitigated membrane fouling through coagulation of macromolecules in the sludge mixture. The average Fe^3+^ concentration in the MBR effluent was 0.23 ± 0.11 mg/L under the synergistic effect of the optimal dosing strategy and adsorption by the contaminated layer of the microfiltration membrane. The effluent Fe^3+^ concentration consistently complied with China’s Environmental Quality Standard for Surface Water (GB 3838-2002), which effectively avoids the risk of secondary pollution caused by over-injection of iron salts. The batch dosing strategy is simple to operate and requires minimal additional equipment, demonstrating potential for application in large-scale MBR projects. The mechanism of controlling iron ion leakage through dosing strategies, as revealed in this study, provides a theoretical basis and methodological framework for MBR applications across various scenarios. For wastewaters of different compositions, parameter optimization is recommended prior to full-scale implementation. Future research should explore the long-term stability and applicability of this strategy in more complex wastewater systems.

## Figures and Tables

**Table 1:** Variations in water quality of effluent from reactors #1 and #2 during 25–75 d of operation.

**No.**	**COD** **(mg/L)**	**NH_3_-N** **(mg/L)**	**TN** **(mg/L)**	**TP** **(mg/L)**	**NO_3_-N** **(mg/L)**	**NO_2_-N** **(mg/L)**
#1	18.31 ± 6.92	0.31 ± 0.39	28.65 ± 6.67	3.60 ± 1.86	28.38 ± 5.77	0.04 ± 0.03
#2	18.38 ± 7.60	0.39 ± 0.37	29.49 ± 7.17	3.33 ± 1.43	29.80 ± 6.26	0.07 ± 0.10

**Table 2:** Molecular weight distribution characteristics of organic matter in the supernatant of mixed sludge from reactors #1 and #2.

No.	Organic Matter	Mn (kDa)	Mw (kDa)	Mz (kDa)	Mw/Mn
#1	P1	70.1	72.8	75.8	1.0
	P2	336.3	378.8	443.2	1.1
	P3	2722.9	4521.1	8496.9	1.7
#2	P1	79.2	85.5	92.6	1.1
	P2	361.0	405.0	474.0	1.1
	P3	4905.8	6649.8	9582.8	1.4

## Data Availability

The original contributions presented in this study are included in the article/Supplementary Material. Further inquiries can be directed to the corresponding author.

## Supplementary Materials

The following supporting information can be downloaded at: https://www.mdpi.com/article/10.3390/membranes15100297/s1, Figure S1: Schematic diagram of continuous flow device; Figure S2: Schematic diagram of MBR set-up; Figure S3: Phosphorus-specific release rate measurement device diagram; Figure S4: Phosphorus-specific absorption rate measurement device diagram; Figure S5: Specific oxygen uptake rate measurement device diagram; Figure S6: Comparison of SOUR between reactors #1 and #2; Table S1: Main water quality indicators for the influent of MBR set-up; Table S2: Related design parameters of O-MBR; Table S3: Routine chemical test items and methods; Table S4: Discharge standard of pollutants for municipal wastewater treatment plant; Text S1: Calculation of Fe dosage based on influent TP; Text S2: Determination of sludge-specific oxygen uptake rate.
