# Supplementary material for: Comprehensive Investigation of Iron Salt Effects on Membrane Bioreactor from Perspective of Controlling Iron Leakage
Source: Membranes (Basel). 2025 Sep 30;15(10):297. doi: 10.3390/membranes15100297 (PMC12566238; doi:10.3390/membranes15100297)
Supplement: Supplementary file 1 [file membranes-15-00297-s001.zip › membranes-3867191-supplementary.pdf]

membranes

Supplementary Materials

Comprehensive Investigation of Iron Salt Effects on Membrane Bioreactor from Perspective of Controlling Iron Leakage

Qiaoying Wang, Bingbing Zhang, Jicheng Sun, Wenjia Zheng, Jie Zhang * and Zhichao Wu

State Key Laboratory of Water Pollution Control and Green Resource Recycling, Shanghai Institute of Pollution Control and Ecological Security, College of Environmental Science and Engineering, Tongji University, Shanghai 200092, China; qywang@tongji.edu.cn (Q.W.); 2331416@tongji.edu.cn (B.Z.); 1631342@tongji.edu.cn (J.S.); 2130558@tongji.edu.cn (W.Z.); wuzhichao@tongji.edu.cn (Z.W.)
* Correspondence: zhangjie130@sina.com; Tel.: +86-21-65980400

Text S1. Calculation of Fe dosage based on influent TP.

The dosage of $Fe_2(SO_4)_3$ was determined based on the Fe/P molar ratio of 1.5, accounting for fluctuations in the influent TP. The calculation procedure is detailed below:

Calculation parameters:

1) Iron content in commercial $Fe_2(SO_4)_3$ reagent = 21-23% (as provided by the supplier).
2) Fe/P molar ratio = 1.5
3) Design influent TP concentration = 4.4±0.6 mg/L (refer to Table S1)

Step-by-step calculation:

1) Target phosphorus removal load (considering water quality fluctuations): 5 mg/L;
2) Fe dosage per unit volume (Fe/P = 1.5): $\frac{5}{31} \times 1.5 \times 56 = 13.55$ mg/L;
3) Dosage of $Fe_2(SO_4)_3$ per unit volume: $\frac{13.55}{0.21} = 64.5$mg/L.

Text S2. Determination of sludge-specific oxygen uptake rate.

The specific oxygen uptake rate (SOUR) was determined as follows: A sample of activated sludge was collected from the reactor and centrifuged at 3000 rpm for 5 minutes. After discarding the supernatant, the residual sludge solids were resuspended in 1 L of phosphate-buffered saline (PBS, pH 7.0). As illustrated in Fig. S5, the mixed liquor suspended solids (MLSS) concentration was adjusted to 2-3 g/L. The following chemicals were then added: 100 mg/L $NaHCO_3$, 120 mg/L CH_3COONa, 76 mg/L NH_4Cl, and 99 mg/L $NaNO_2$. The mixture was aerated via a sand core diffuser until the dissolved oxygen (DO) concentration reached 6-8 mg/L. Aeration was then stopped, the bottle was sealed, and magnetic stirring was initiated. The DO concentration was monitored periodically with a DO meter until it decreased below 1.0 mg/L.

Table S1. Main water quality indicators for the influent of MBR set-up.

	**COD**	**NH_3-N**	**TN**	**TP**	**SS**
Water quality (mg/L)	350 ± 94	32.8 ± 4.6	41.6 ± 3.7	4.4 ± 0.6	75.2 ± 15.1

Table S2. Related design parameters of O-MBR.

**Parameters**	**Sludge age (d)**	**Volume (L)**	**HRT (h)**	**Sludge concentration (g MLSS/L)**	**Excess sludge (kg MLSS/d)**	**Aeration intensity ($m^3/m^2 \cdot min$)**
Values	20	10.5	4.3	12.0	0.01	1.0

Table S3. Routine chemical test items and methods.

**Analyze the project**	**Analytical method or analytical instrument**	**Method source or manufacturer**
COD	Potassium dichromate	APHA
MLSS	gravimetric method, dried at 103-105°C for 2 hours	105°C drying and weighing method
	Partech 740 Portable Sludge Concentration Meter	Partech, UK
MLVSS	Gravimetric method, drying at 600°C for 2 hours	600°C drying and weighing method
NH_3-N	Nano reagent colorimetric method	APHA-4500-NH_3
NO_3^--N	Ultraviolet spectrophotometry	APHA-4500-NO_3^-
NO_2^--N	N-(1-Naphthyl)-ethylenediamine photometric method	APHA-4500-NO_2^-
TN	Alkaline potassium persulfate digestion by ultraviolet spectrophotometric method	APHA-4500-N
TP	Molybdate amine spectrophotometry	APHA-4500-P
Total alkalinity	titration	APHA
Dissolved oxygen	electrochemical probe method	Hash, America
pH	glass electrode method	Hash, America
Sludge particle size	Mastersizer 2000	Malvern, UK

Note: The instrument model of the UV-Vis spectrophotometer used was TU–1810.

Table S4. Discharge standard of pollutants for municipal wastewater treatment plant. (GB 18918-2002).

**Water quality indicators（mg/L）**	**COD_{Cr}**	**BOD_5**	**TN**	**NH_3-N**	**TP**
Grade A	50	10	15	5（8）	0.5

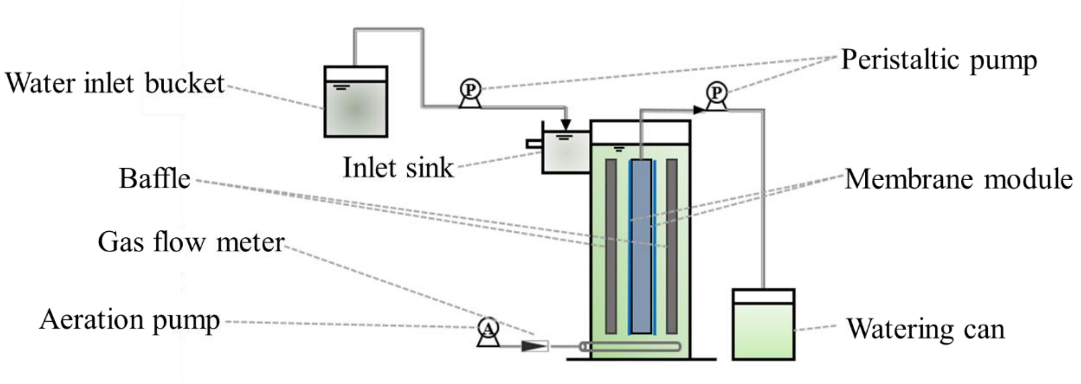

Figure S1. Schematic diagram of continuous flow device.

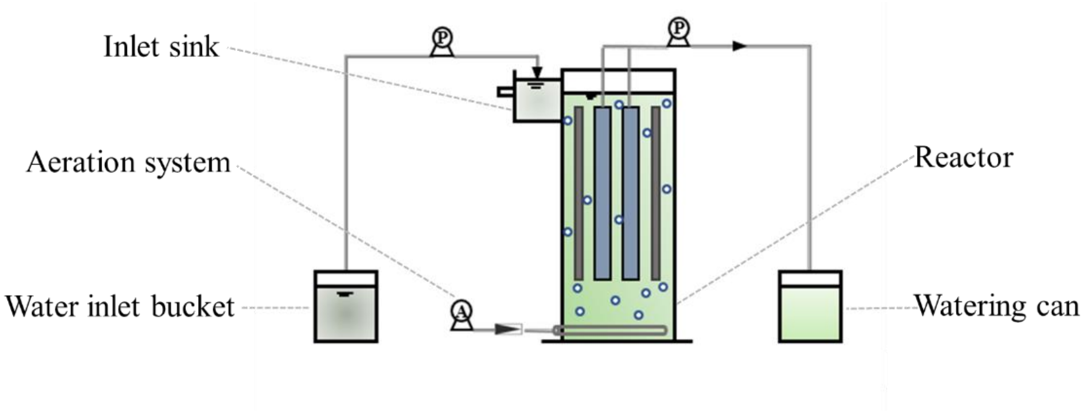

Figure S2. Schematic diagram of MBR set-up.

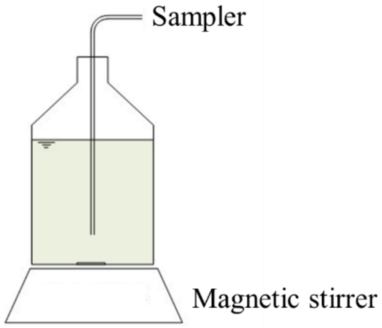

Figure S3. Phosphorus-specific release rate measurement device diagram.

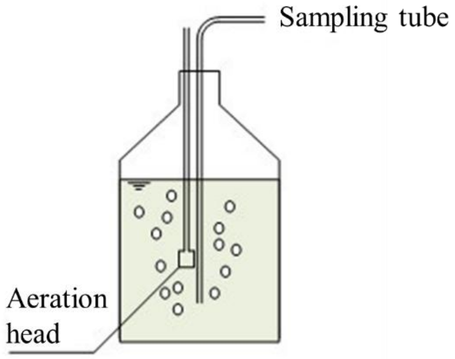

Figure S4. Phosphorus-specific absorption rate measurement device diagram.

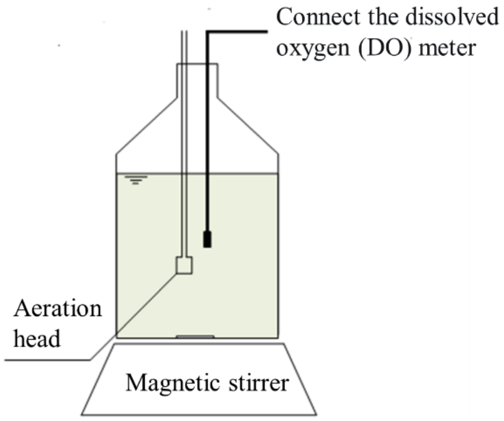

Figure S5. Specific oxygen uptake rate measurement device diagram.

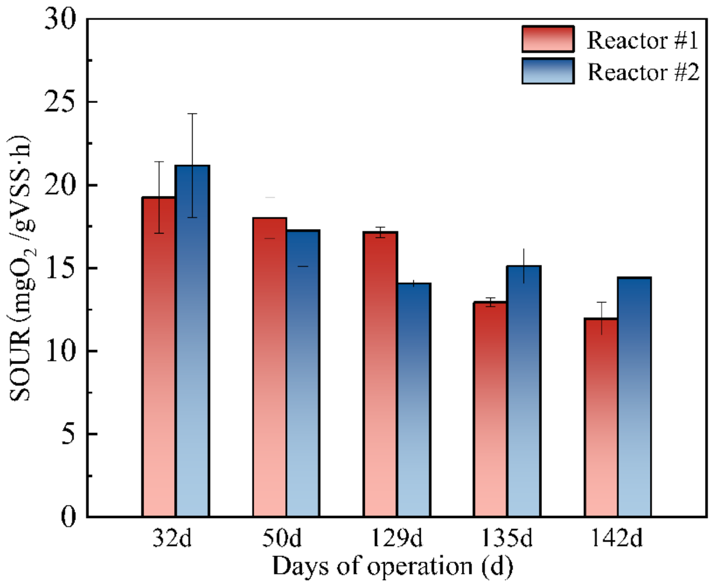

Figure S6. Comparison of SOUR between reactors #1 and #2.
